# Needs of family members of patients in intensive care and their perception of medical communication

**DOI:** 10.5935/2965-2774.20230374-en

**Published:** 2023

**Authors:** Augusto Garcia de Cezar, Flávia Del Castanhel, Suely Grosseman

**Affiliations:** 1 Universidade Federal de Santa Catarina, Florianópolis (SC), Brazil

**Keywords:** Communication, Family, Critical illness, Professional-family relations, Intensive care units, Surveys and questionnaires

## Abstract

**Objective:**

To understand the perception of medical communication and needs of family members with loved ones in intensive care.

**Methods:**

The study was mainly qualitative and exploratory, with thematic analysis of comments made by 92 family members with loved ones in intensive care units when answering in-person interviews comprising the Quality of Communication Questionnaire (QoC) and open-ended questions about their need for additional help, the appropriateness of the place where they received information, and additional comments.

**Results:**

The participants’ mean age was 46.8 years (SD = 11.8), and most of them were female, married and had incomplete or completed elementary education. The following themes were found: perception of characteristics of medical communication; feelings generated by communication; considerations about specific questions in the QoC; family members’ needs; and strategies to overcome needs regarding communication. Characteristics that facilitated communication included attention and listening. Characteristics that made communication difficult included aspects of information sharing, such as inaccessible language; lack of clarity, objectivity, sincerity, and agreement among the team; limited time; and inadequate location. Feelings such as shame, helplessness, and sadness were cited when communication was inadequate. Family members’ needs related to communication included more details about the loved one’s diagnosis, prognosis, and health condition; participation in decisionmaking; and being asked about feelings, spirituality, dying and death. Others were related to longer visitation time, psychological support, social assistance, and better infrastructure.

**Conclusion:**

It is necessary to enhance medical communication and improve hospital infrastructure to improve the quality of care for family members.

## INTRODUCTION

Having a loved one hospitalized in the intensive care unit (ICU) has a great emotional and psychic impact on family members. This context can generate a “whirlwind of feelings”^(1)^ and even cause psychiatric disorders such as anxiety, posttraumatic stress, and depression in some family members.^(1,2,3,4)^

Several factors can influence family members’ suffering and stress. These include the loved one’s health condition in itself; the fear of their loved one dying; significant changes in the dynamics of personal life resulting from their loved one’s illness; the ICU environment, which is typically noisy, seems impersonal and contains frightening equipment, such as monitors and mechanical ventilators; and the environment of the waiting room or hospital corridor, where family members wait for news about their loved one’s health.^(1,2,3)^

The anguish caused by waiting for information makes communication with the physician a decisive moment that may reduce or increase family members’ uncertainties. In the context of Brazilian ICUs, this is still usually done by means of medical reports and, less frequently, in family conferences. One of the functions of this communication is to clarify doubts regarding diagnosis, treatment, and prognosis. An additional function is for the physician to obtain information about the loved ones when they are not able to communicate and manifest their perspectives, needs, values, and desires. ^(1,2,5,6,7)^ In the latter case, family members become the patient’s “voice,” and some responsibilities fall on them, such as participating in the therapeutic decisionmaking process, which can cause additional suffering.^(1,2,3,8)^

It is well established that in communication between physicians and family members, both the patient and their family members should be considered members of the team.^(9)^ Physicians should welcome, build and maintain rapport with family members; speak objectively, in clear language, without using technical terms; encourage them to participate actively in meetings, asking open-ended questions and providing room for them to express their perspectives, doubts, and feelings; listen carefully, responding to their emotions with empathy and compassion; and clarify their doubts, so that they have clarity about the situation and feel more confident in participating in decision-making processes whenever they wish to do so and whenever it is necessary. ^(2,3,8)^ In spite of the importance of communication, studies have shown that some family members do not fully understand their loved ones’ diagnosis, care plan, and prognosis, and they feel that professionals communicate impersonally without showing empathy or compassion. ^(1,2,5,7)^

Knowledge about the perception of family members whose loved ones are in intensive care regarding the quality of medical communication, as well as knowledge about their needs, is essential to evaluate the level of care being provided and to help promote measures aimed at reducing potential damage to the health of patients and their family members.

Curtis et al. developed an instrument called the Quality of Communication Questionnaire (QoC) to assess the quality of medical communication by patients with chronic diseases at the end of life, containing items related to general communication and end-of-life communication.^(10,11)^

The QoC was translated and cross-culturally adapted to Brazilian Portuguese in 2017,^(12)^ and subsequently validated in 2021.^(13)^ Furthermore, it was adapted for family members, translated into Brazilian Portuguese and validated with permission from the authors of the original scale.^(14)^ During the interviews with family members, when requesting their evaluation of each QoC item, many of them made spontaneous comments to justify their ratings. These comments were recorded in writing by the interviewer. Spontaneous comments appeared in the study by Russel in Australia,^(15)^ as well as in the study by the authors of the present article when applying the QoC to patients.^(16)^

Considering the importance of family members’ viewpoints and perceptions of the construction of knowledge that can support future actions to promote the quality of care, the objective of this study was to understand the perception of medical communication and the needs of family members with loved ones in intensive care.

## METHODS

This study’s design was mainly exploratory and qualitative, but it has a quantitative approach to characterizing the participants. It is part of a larger project for validation of the QoC, approved by the Ethics Committee of the *Universidade Federal de Santa Catarina* under number 77721917.8.0000.0121, with the permission of the authors of the original scale.

Written informed consent for family members’ information to be published was provided by the family members.

The study participants were family members of patients hospitalized in the ICU in four public hospitals in southern Brazil.

The selection was made by convenience, inviting eligible participants who were present in the study location at the time of the researcher’s visit.

The inclusion criteria were being a family member of a patient hospitalized in the ICU for at least 24 hours, being 18 years or older, and being Brazilian.

The exclusion criterion was having difficulty communicating due to being emotionally overwhelmed (as observed by the nursing team).

The invitation to participate in the study was made in person after explaining the study objectives, the form of data collection, the possibility of publishing it anonymously, and all ethical precepts. The family members who agreed to participate in the study received two copies of the free and informed consent form to read and sign, keeping one of the copies.

### The Quality of Communication Questionnaire

The QoC contains 13 questions divided into two subscales, one on general communication (Items 1 to 6) and the other on end-of-life communication (Items 7 to 13).^(10,11)^ The subscales can be used separately or together, depending on the aspect of communication being studied. The version of the QoC adapted for family members is exhibited in appendix 1.

**Appendix 1:** Brazilian version of Quality of Communication Questionnaire for family members and its back-translation

Brazilian version of QoC for family members	Back-translation of the Brazilian version of QoC for family members
*Gostaríamos de saber, o mais detalhadamente possível, o quanto o(a) médico (a) que cuida dos problemas de saúde de seu(ua) ente querido (a) é bom(a) em falar com o(a) senhor(a) sobre a doença dele(a) e os tipos de cuidados que ele(a) gostaria de receber se ficasse pior ou doente demais para responder por si mesmo(a). Sabemos que muitas pessoas têm grande admiração por seus (uas) médico(a)s. Para nos ajudar a melhorar a comunicação entre médico(a)s e familiares, por favor, seja crítico(a).*	We would like to know, in as much detail as possible, how good the doctor taking care of your loved one health problems is good in talking with you about his or her illness and the types of care he or she would want if he or she became sicker or too sick to speak for himself/herself. We know that many people think very highly of their doctors. To help us improve communication between doctors and family members, please be critical.
*Enunciado: Ao falar com o(a) médico(a) sobre questões importantes, como seu(ua) ente querido(a) ficar muito doente, o quanto ele(a) é bom/boa em:*	Enunciate: When talking with Doctor important issues, such as your loved one becoming very ill, how good he or she is in:
1. *Usar palavras que o (a) senhor (a) consiga compreender*	1. Using words that you can understand.
2. *Olhar em seus olhos.*	2. Looking you in your eyes.
3. *Responder a todas as dúvidas sobre a doença de seu(ua) ente querido(a).*	3. Answering all questions about the illness of your loved one.
4. *Ouvir o que o(a) senhor(a) tem a dizer*	4. Listening to what you have to say.
5. *Preocupar-se com o(a) senhor(a) como pessoa.*	5. Caring about you as a person.
6. *Dar atenção plena ao(à) senhor (a).*	6. Giving you full attention.
7. *Falar sobre seus sentimentos se acaso seu(ua) ente querido (a) piorar.*	7. Talking about your feelings if your loved one gets sicker.
8. *Dar detalhes sobre a condição de seu(ua) ente querido(a), se acaso ele(a) vier a piorar.*	8. Giving details about your loved one’s condition if he or she gets sicker.
9. *Falar sobre quanto tempo seu(ua) ente querido(a) tem de vida.*	9. Talking about how long your loved one might have to live.
10. *Falar sobre como o morrer poderia ser.*	10. Talking about how dying might be.
11. *Envolver o(a) senhor(a) nas discussões do tratamento para o cuidado de seu(ua) ente querido(a).*	11. Involving you in discussions about the treatment of your loved one.
12. *Perguntar sobre coisas importantes da vida de seu(ua) ente querido(a).*	12. Asking about important things in life of your loved one.
13. *Perguntar sobre suas crenças espirituais ou religiosas.*	13. Asking you about spiritual and religious beliefs.

QoC - Quality of Communication Questionnaire.

Source: Authors, 2021.

### Data collection

The data collection instrument was a structured questionnaire with sociodemographic variables (age, sex, level of education, marital status, relationship with the patient); cause of hospitalization in the ICU of family members’ loved ones; the QoC and family members’ spontaneous comments to justify their ratings in each QoC item during the interview, which were recorded in writing by the interviewer; and the following three open-ended questions: “How was your perception of the place where you received the medical report?”, “What type of complementary assistance would you like to receive?”, and “Are there any other questions that are not included in this questionnaire that you would like to be addressed?”.

The main focus of this study is on qualitative data. The quantitative aspects of QoC are not approached in the present study because they have already been published.^(14)^

The questionnaires were administered by in-person interview either in the waiting room, when it was possible to maintain privacy, or in a reserved place in the hospital.

Data collection was conducted by a previously trained researcher before or after the family member visited the hospitalized loved one. Data collection occurred between October 24, 2015 and August 2, 2016, as well as between August 15, 2018 and October 25, 2019.

The interviews lasted at least one hour because family members took advantage of the opportunity to talk about their experiences, needs, and perceptions, to which the researcher listened attentively and recorded their responses.

### Data analysis

The data were entered in a database using Statistical Package for the Social Sciences (SPSS), version 26.0.

Analysis of sociodemographic data and the causes of hospitalization of family members’ loved ones was carried out using descriptive statistics: Student’s t test (t) to compare two groups in relation to continuous variables, and Pearson’s chi-square test (χ2) for two groups of categorical variables. The null hypothesis was rejected if its probability was less than 0.05.

Analysis of qualitative data was thematic, starting with a reading of the reports for familiarization, without marking the text, followed by the identification of units of meaning (words or terms), units of context (search for contexts interrelated to the units of meaning), and nuclei of meaning (themes), relating and interrelating the previous units.^(17)^

Participants’ comments to illustrate some units of context are identified in the results with F and a number for females (for example, F1) and M and a number for males (for example, M1).

## RESULTS

The mean age of the 92 family members participating in the study was 46.8 years (standard deviation - SD = 11.8), with no difference by gender [t(90) = -0.17; p = 0.87]. Other sociodemographic characteristics of the participants are exhibited in table 1. There were more female than male participants [χ²(1) = 14.1; p &lt; 0.001].

**Table 1:** Sociodemographic characteristics of 92 family members participating in the study

Characteristics	n (%)*
Gender	
Female	64 (69.6)
Male	28 (30.4)
Level of education	
Incomplete elementary education	23 (25.0)
Completed elementary education	27 (29.3)
Incomplete secondary education	9 (9.8)
Completed secondary education	16(17.4)
Incomplete tertiary education	11 (12.0)
Completed tertiary education	6 (6.5)
Marital status	
Married	55 (59.8)
Divorced	1 (1.1)
Civil partnership	24(26.1)
Widow	4 (4.3)
Single	8 (8.7)
Relationship with the patient	
Daughter/son	32 (34.8)
Spouse	26 (28.3)
Sibling	18(19.6)
Parent	9 (9.8)
Brother-in-law/sister-in-law	2 (2.2)
Cousin	2 (2.2)
Uncle/aunt	2 (2.2)
Son-in-law/daughter-in-law	1 (1.1)

* The total percentage is 102% due to rounding percentages to one decimal place.

The causes of hospitalization of the participants’ loved ones as reported by them are displayed in table 2.

**Table 2:** Causes of hospitalization of loved ones admitted to the intensive care unit of 92 family members participating in the study

Cause of the loved one’s hospitalization	n (%)
Cardiovascular: n = 25(27.18%)	
Abdominal aortic aneurysm	2(2.17)
Unspecified coronary surgery, myocardial revascularization surgery, or angioplasty	7(7.61)
Pulmonary thromboembolism	3 (3.26)
Congestive heart failure	1 (1.09)
Catheterization	5 (5.43)
Aortic valve implantation or replacement	4 (4.35)
Acute myocardial infarction	1 (1.09)
Intracardiac tumor resection	1 (1.09)
Heart problem without a specified cause	1 (1.09)
Respiratory or noncardiovascular thoracic: n = 18 (19.55%)	
Pneumonia	9 (9.78)
Lung biopsy or resection	2(2.17)
Influenza type A	1 (1.09)
Asthma	2(2.17)
Respiratory failure	2(2.17)
Chronic obstructive pulmonary disease	2(2.17)
Gastrointestinal/abdominal: n = 6 (6.53%)	
Peptic ulcer	1 (1.09)
Partial enterectomy	1 (1.09)
Cirrhosis	1 (1.09)
Intestinal hemorrhage	1 (1.09)
Intestinal cancer	2(2.,17)
Neurological: n = 9 (9.78%)	
Subarachnoid hemorrhage	3 (3.26)
Stroke	2(2.17)
Traumatic brain injury	4 (4.35)
Renal: n = 3 (3.26)	
Chronic kidney disease	2(2.17)
Nephrectomy	1 (1.09)
Other causes: n = 31 (33.70%)	
Sepsis	16(17.39)
Lymphoma	1 (1.09)
Leptospirosis	1 (1.09)
Exogenous intoxication	3 (3.26)
Polytrauma	3 (3.26)
Bone marrow transplantation	2(2.17)
Unspecified postsurgical cause	2(2.17)
Zika virus infection	1 (1.09)
Systemic lupus erythematosus	1 (1.09)
Diabetic ketoacidosis	1 (1.09)
Total	92(100.0)

The following themes were found: perception of characteristics of medical communication; feelings generated by communication; considerations about specific questions in the QoC; family members’ needs; and strategies to overcome the perceived needs regarding communication.

**Figure 1:**
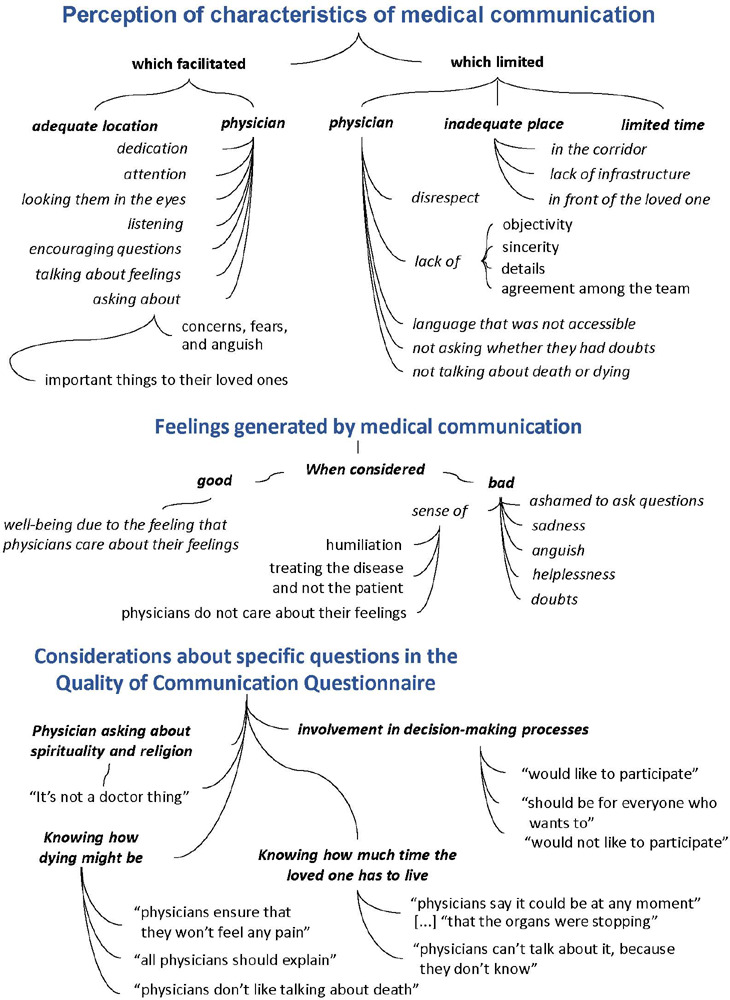
Perception of medical communication characteristics, feelings generated by them, and considerations about specific questions in the Quality of Communication Questionnaire

Figure 1 displays the theme “medical communication”, as well as the units of context and meanings found.

The characteristics that facilitated communication with physicians with family members included an adequate location for the conversation and the physicians dedication, attention, eye contact, listening skills, expression of their feelings, questions about things important to their loved one and about their concerns, fears and anguish.

The characteristics that limited communication were diverse. One was the low intelligibility regarding what physicians were talking about, with one family member exclaiming that physicians “should not use expressions that we do not understand” (F01). This fact was further aggravated when family members felt that the physicians spent little time talking to them, making them feel like the physicians were “in a hurry”, “not talking to them enough” and leaving them “without understanding anything” (M01). They also mentioned their feelings of shame when “asking what those ‘bad words’ mean” (F02), referring to medical terminology used during communication.

The lack of objectivity and of details about the loved one’s health condition was well exemplified by one of the family members: “They only answer: ‘We are taking good care, everything’s going to be okay’“ (F03). Regarding participation in decision-making, one family member expressed that, as they did not understand anything, “the doctor takes care of it all alone and doesn’t ask me anything” (F04). Some family members mentioned their perception of physicians’ lack of sincerity, as illustrated in the following statement: “Sometimes I feel like the physicians feel sorry for me and hide the fact that my husband doesn’t have a chance of surviving” (F05).

The discrepancies between the information provided by the team members was also highlighted, for example, in the following statement: “[...] one physician says the state is terminal and there is nothing else to be done; another says that not everything is lost. They should all reach a consensus [...]” (F06).

The place where the information was provided; either in front of the loved one or in the corridor, without a space reserved for the conversation; was considered another limiting aspect of communication by some family members. Others, however, mentioned that, provided that the necessary information was communicated, the location was not important.

Regarding attitudes, some family members reported that certain physicians did not seem to care about them or their loved ones, and they had the sense that they were “more committed to treating the disease and not the patient as a whole” (F07) or “mostly worried about curing and not about caring” (F08).

One feeling expressed was anguish while waiting for information, for instance, while the mother of Participant M02 was being transferred to the ICU after heart surgery, the participant reported that he was waiting “in a state of immense affliction, and no one came, not even to say that everything was fine” and that this was “totally inhumane” because “for someone who is waiting, minutes turn to hours”.

Attitudes of disrespect and lack of sensitivity on the part of physicians were reported, not only affecting the relationship between the physician and the family member but also generating, for the family member, feelings like helplessness, sadness, a sense of humiliation, and doubt regarding the quality of the treatment provided, as illustrated in the following statement:

[...] after my mother’s surgery, I went after the evening visiting hours were over, but I had the nurse’s permission. The doctor humiliated me in front of the team, saying that it wasn’t time to give out information about the patient’s clinical status and that I would have to wait for the medical report. After that, since I know that he’s the one who’s giving the medical report, I don’t stay to receive the information. [...] the situation generated a feeling of helplessness and sadness for having to leave my mother in the hands of an insensitive person, generating doubts about the treatment she would be receiving, since that physician treated me so disrespectfully (M02).

Family members’ considerations about specific questions in the QoC, such as participation in decision-making, were diverse. While some did not want to or even believe that they should participate in decision-making, others thought that this participation “should be for everyone who wants to” (M03), and participant M04 expressed the desire to participate in all “meetings” about their spouse’s health status.

Regarding the physician’s approach to family members’ feelings, if the loved one’s health condition worsened, some family members mentioned that the physicians did not ask about their feelings, and others said they turned to other professionals. Participant F09 mentioned talking to nurses about their fears, and another commented that “the psychologist is the one who talks about ‘those topics’“ (F05).

In relation to spiritual and religious beliefs, one family member said that they did not know whether asking about beliefs and spirituality was “a doctor thing” (F10).

Regarding the question as to whether the physician talked about how long their loved one might have to live, while relative M05 commented that the doctors said that it could be at any moment and that the “organs were shutting down”, another said that “physicians could not talk about this subject because they did not know” (F06).

Regarding physicians talking about death and dying, one family member mentioned that the physicians did not like “talking about death” (F11), and Participant F12 said that physicians ensured that the patient would not feel any pain. However, one participant considered that all physicians should explain “the details before death” (F13).

**Figure 2:**
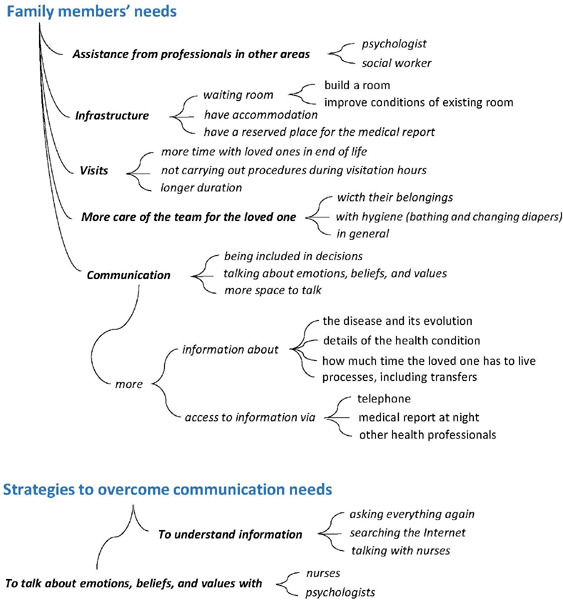
Needs and strategies to overcome the communication needs of family members with loved ones hospitalized in the intensive care unit

Figure 2 displays the categories, units of context, and meanings of family members’ needs, as well as their strategies to overcome communication needs.

The needs felt by family members were diverse. Those related to communication included wanting more information about the loved one’s health condition, with more details, “not just the same thing, ‘unstable, not reacting, very bad’“ (M05). Participant M06, family member of a patient colonized by *Klebsiella pneumoniae carbapenemase,* wanted to know more details about the risks of contamination.

Lack of information when a loved one was transferred from the ICU to the ward was also reported by a family member who went to the hospital to visit him in the ICU and found that he was no longer there. In their words, “There is a total lack of organization regarding information. If I had known sooner, I’d have come prepared to stay as a companion” (F13).

Regarding access to information, the need of a medical report during the night for family members who work during the day was mentioned, as well as, of the possibility of the medical report being provided by nursing professionals at night and providing information by telephone.

Regarding visitation, several family members suggested longer visitation times, as they felt the need to stay more than 30 minutes (duration of the visit) with their loved ones. They also suggested that they should be able to stay longer, depending on the health condition or if the loved one was at the end of life. Furthermore, they suggested that procedures be performed at a time that did not interfere with the visits.

The need for support from other professionals from the team was mentioned by some family members who required assistance from a social worker for issues such as social security benefits, as well as psychological support during the hospitalization period.

Regarding infrastructure, the place where the medical report was provided was also considered inappropriate among family members with loved ones admitted to hospitals that did not have a waiting room because they waited for the visit and received the medical report while standing in the corridor. Therefore, they mentioned the need for a waiting room and a place reserved for the physician to deliver the medical report. Furthermore, they mentioned the need to improve the conditions of some existing rooms because some family members felt that the waiting room was not “decent” (F14). However, some family members underscored that they did not consider the location important, provided that the medical report they received met their information needs. They also reported the need for accommodation for family members who did not live in the city and who could not afford a hotel or who lived far from the hospital. Moreover, according to family members, accommodation would make it possible for family members to take turns accompanying patients.

The need for greater care and “more attention from the nursing team” (M08) or the team as a whole was also mentioned. Participant F15 mentioned that she would like the team to be more careful with their loved one’s belongings because their mother’s dental prosthesis had been lost. Another commented that “the technician said she was not going to make an effort alone to take care of hygiene” (M08) and that the conflicts with the team left the loved one and the family member distressed, exclaiming that, “After all, they aren’t doing it as a favor!” (M08). Moreover, one family member commented that her brother needed to have his diaper changed, and up to the end of the shift, he had not been cleaned.

Family members cited some strategies they used to overcome communication needs. One of them was to talk about their feelings with the nursing team because they were attentive professionals. Other strategies were developed to clarify their doubts about the information shared, which included asking the physician “everything all over again” (F16), asking the nursing team about what they did not understand, and seeking further information on the internet when they did not understand the words used by the physician.

## DISCUSSION

Our study showed that some physicians were attentive, made eye contact, listened to the family members, and asked about their feelings and things important to the life of their loved one, thus communicating effectively and developing a positive rapport with them.

This finding is in line with that of an integrative review conducted in 2018 on the satisfaction of family members of patients hospitalized in the ICU, which found that professionalism, competence of the team, and respect for the family and the patient were related to better experiences for family members.^(6)^

On the other hand, our study also demonstrated many characteristics that limited communication, such as sharing information in language that was not accessible; with little clarity and objectivity; without details and without clarification of family members’ doubts, performed in a “hurried” manner without sufficient time and with disagreements within the team. Along with these perceptions, the feeling that the physician was not being sincere as well as feelings of shame related to asking questions contributed to many family members not understanding their loved ones’ clinical diagnosis and prognosis. The reported feelings showed how inadequate physician communication and distanced attitudes aroused negative feelings that increased family members’ suffering, including anguish caused by delays in receiving information, feelings of helplessness and sadness, and the sense of humiliation caused by disrespectful attitudes from medical professionals. These attitudes even caused some family members to feel that some physicians were insensitive and primarily concerned with curing the patients’ diseases rather than taking care of them as people and that they therefore did not care about their feelings.

These findings are similar to those of a systematic review published in 2017 on end-of-life care in the ICU, which showed a high percentage of family members who did not fully understand their loved one’s diagnosis, prognosis, and care and who received contradictory information.^(2)^ The importance of sincerity in sharing information was emphasized in this and other studies.^(2,5,6)^ Some studies included in the review conducted in 2018 reported physicians being rude, aggressive, insensitive, and lacking interpersonal skills.^(6)^ In some studies included in the 2017 review, the family members perceived that physicians did not consider their feelings, did not show empathy or compassion, did not take their presence into consideration, and shared information about their loved ones in an impersonal manner.^(2)^ It has been well established that effective communication, support, care, and rapport with both the patient and their family members, as well as clarification of their doubts, are necessary to increase family members’ confidence and participation in decision-making processes and to reduce and/or prevent their suffering. ^(2,3,8)^

In our study, family members expressed the need to receive more information about their loved ones’ disease, prognosis, and clinical conditions on a daily basis in a more detailed manner. This finding aligns with other studies.^(1,5,7)^ A quantitative study with 40 family members highlighted the importance of implementing measures to provide accurate information on the patient’s prognosis, the care provided, and the ICU routines, in addition to recommending ways for the family members to contribute to patient care.^(5)^

Involving family members with the team’s treatment process has also been recommended.^(9)^ Their inclusion in medical visits allows them to be heard, to clarify their doubts, to participate in therapeutic discussions and to speak about patients’ values.^(2)^ Additionally, when they are encouraged by physicians to speak more in family conferences, they feel more satisfied with the care provided.^(8)^

Regarding the level of family members’ involvement in decision-making, whereas some would like to participate— and one even mentioned that they would like to attend team meetings on the health of their loved one—others reported that they did not wish to participate.

Other studies have also shown this difference in whether family members wish to be involved in decision-making. Therefore, it is essential for physicians to evaluate the degree of involvement that the family member wishes to have in the decision-making process^(8)^ because they are usually not asked whether or not they prefer to be included. ^(2)^ While some studies have shown increased satisfaction of family members with greater participation in decision-making processes,^(1,2,6,7,8)^ others have shown that this is not always the case. A study with family members of patients from 78 ICUs found that half of them did not want to participate and that, among those who participated, there was greater emotional stress.^(18)^ A hypothesis for this increased stress when participating in decision-making is that family members did not receive enough information to sufficiently understand their loved one’s diagnosis, therapeutic possibilities, and prognosis in order to make them feel confident and safe in decision-making.

Another need reported by family members in our study was an adequate waiting room or improvement in the conditions of the existing room, as well as a place reserved for the medical report in order to avoid communication in corridors. Furthermore, in our study, family members mentioned the need for accommodations where family members who lived far away from the hospital or in other cities could rest. Other studies have reported family members’ discomfort regarding the place where the information is shared.^(1,2,6)^ Adequate waiting rooms can reduce the risk of anxiety, posttraumatic stress, and depression in the family,^(3)^ and single rooms at the end of life are highly valued by family members.^(6)^

Regarding the strategies adopted by family members to overcome their communication needs, one strategy found in our study was to reach out to the nursing team to clarify their doubts and talk about their feelings.

Different studies have highlighted nurses as a great ally to family members, who classified nurses as their main source of information and important emotional support.^(1,2,3,5,6,7)^ An action usually performed by nurses to reduce family members’ uncertainties and emotional stress is to give a tour of the ICU environment and demystify it, explaining the procedures, devices, and dynamics.^(1,2,6)^ Therefore, the quality of nursing care is an essential component for family members’ satisfaction.^(2,6)^

On the other hand, in our study, although the nursing team was indicated as fundamental for emotional support and for clarifying family members’ doubts, some reported that there were members of this team who paid little attention and were less committed to their loved ones, citing, as examples, loss of their belongings and problems related to hygiene. Studies have shown that when there is a lack of trust in the nursing team, family members become more vigilant.^(1)^ Furthermore, inappropriate conversations on the part of the team, especially the nursing team, were detrimental to family members’ well-being.^(6)^

Although many family members wanted to talk about their beliefs and spirituality, there were participants who believed that this was not the physician’s job. The literature, however, has shown that care for family members’ spiritual needs is extremely important and is associated with greater satisfaction with the decision-making process at the end of life,^(8)^ as it helps family members deal with the death of their loved one, reduces their sense of guilt, and reduces negative psychological impacts.^(2,6)^

This also occurred when asking about family members’ feelings in cases when the loved one got worse, especially in cases related to death and dying. Although some family members did not consider it to be the physician’s job to ask about these topics, many mentioned that they wanted more details on these subjects, and some commented that it seemed that physicians did not like to talk about death or about how much time their loved one had to live.

Studies have demonstrated physicians’ difficulties in communicating with patients and their family members at the end of life.^(5,6,7)^ A review published in 2020 analyzing the perspective of family members with loved ones in end-of-life care in an ICU found that inadequate care, lack of support, and failure on the part of health professionals to provide updated information on loved ones’ health conditions cause emotional distress for family members.^(7)^ On the other hand, studies have demonstrated that providing written information helps family members know what to expect from the end-of-life process, brings a greater sense of control, and better prepares them for the death of a loved one.^(8,19)^

Additionally, the family members in our study reported the need to spend more time with their loved ones during visits, especially towards the end of life. The importance of this closeness to positive experiences for family members has been indicated in several systematic reviews.^(1,3,7,8)^ Making visiting hours more flexible is a change that can decrease the risk of post-intensive care syndrome in the family and increase their satisfaction with care.^(1,3)^ When associated with the active participation of the family member in the care of the patient, respect for cultural values and emotional and spiritual support strengthens the bond with the team and facilitates decisionmaking.^(7)^ Furthermore, family members value the opportunity to be present at the moment of their loved one’s death.^(8)^

Although it was not found in our study, other studies have demonstrated the influence on family members of factors related to the ICU, such as the volume of sounds, the brightness of lights, and the cleanliness of spaces.^(1,2,6)^

The limitations of our study included the selection of participants according to convenience and the fact that not all family members made spontaneous comments about all QoC items, even though all of them answered the open-ended questions.

Nevertheless, our study has demonstrated the need to improve various aspects of communication and professionalism among some physicians who work in intensive care. These include considering family members as part of the team; welcoming them in an appropriate place with sufficient time; showing respect, attention, commitment, empathy, and sensitivity; listening to their needs, doubts, concerns, expectations, feelings, beliefs, and spirituality; sharing information using clear and accessible language with details about their loved one’s clinical conditions, diagnosis, prognosis, and anything else they express that they wish to know; including them in the decision-making process; and discussing death and the process of dying, whenever possible and desired by the family. In the institutional sphere, the availability of adequate environments for family members is a demonstration of appreciation and respect for them. Therefore, it is essential to have appropriate spaces for waiting, meetings, and family conferences, in addition to a place where family members can sleep or stay whenever possible.

## CONCLUSION

This study aimed to understand the perception of family members whose loved ones were hospitalized in the intensive care unit regarding medical communication, as well as their needs.

Diverse characteristics of communication were considered by family members as facilitating or limiting communication, showing the importance of greater preparation of physicians and other team members to communicate effectively with one other, with patients, and with their family members, considering their informational, emotional, and spiritual needs. Furthermore, it was possible to observe the importance of caring for the patient—family relationship. Promoting infrastructure that offers comfort and privacy and demonstrates appreciation and respect for those receiving and providing care; facilitating access to information and ensuring comprehension; making visiting hours more flexible; ascertaining the desired degree of involvement in the decision-making process; and talking about death and dying are important aspects that need to be addressed.

It is hoped that the results of this study will encourage the development of strategies aimed at improving the communication of physicians and teams with one another and with family members, in addition to strategies to meet family members’ needs, thus promoting well-being and reducing emotional suffering.
